# Differential cytokine gene expression profiles in the three pathological forms of sheep paratuberculosis

**DOI:** 10.1186/1746-6148-3-18

**Published:** 2007-08-14

**Authors:** Jennifer A Smeed, Craig A Watkins, Susan M Rhind, John Hopkins

**Affiliations:** 1 Centre for Infectious Diseases, Royal (Dick) School of Veterinary Studies, Summerhall, Edinburgh, EH9 1QH, UK; 2 Moredun Research Institute, International Research Centre, Pentlands Science Park, Penicuik, Midlothian EH26 0PZ, UK; 3 Division of Veterinary Clinical Studies, Royal (Dick) School of Veterinary Studies, University of Edinburgh, Easter Bush Veterinary Centre, Midlothian EH25 9RG, UK

## Abstract

**Background:**

Johne's disease is a chronic inflammatory disease of the gut caused by infection with *Mycobacterium avium *subspecies *paratuberculosis *(MAP). Symptoms include wasting, diarrhoea, loss of condition and eventual death. Three forms of Johne's disease have been described in sheep – paucibacillary, multibacillary and asymptomatic. The paucibacillary form is characterized by an inflammatory, Th1-type immune response. The multibacillary form of the disease, which disseminates the infection, is characterized by macrophage infiltration mediated by a Th2-type immune response, and asymptomatic animals have no clinical symptoms or pathology but are infected with MAP. What determines these three forms of the disease is unknown. To further understand these differences, we used real-time RT-PCR to compare the expression of thirteen cytokine and cytokine-related genes in ileal tissue from sheep with the three forms of the disease.

**Results:**

Three pathological forms of sheep paratuberculosis were defined on the basis of histopathology, cytochemistry (Zeihl-Neelsen) and IS900 PCR. Paucibacillary lesions have largely T cell and eosinophil infiltration and are ZN negative; multibacillary lesions have macrophage infiltration and large numbers of acid-fast bacteria. The pauci- and multibacillary forms are linked to the differential expression of IFNγ and IL-10 respectively. In addition the increased levels of the proinflammatory cytokines (IL-1β and TNFα), IL-8, IL-18 and TRAF-1 in both diseased forms is indicative of persistent inflammatory lesions. No changes were seen in IL-1α in any sheep ileum tissues. Asymptomatic animals are IS900+ with normal histology but have significantly decreased levels of IL-18 and increased levels TNFα.

**Conclusion:**

We have quantified the expression levels of thirteen cytokine and cytokine related genes in three forms of ovine paratuberculosis using real-time PCR analyses and confirm that sheep pauci- and multibacillary disease are linked to type 1 and type 2 T cell responses respectively. The expression patterns of other cytokines shows that both disease forms have an inflammatory aetiology but that the central role for IL-1α in bovine paratuberculosis is not seen in the sheep infection. Asymptomatic animals are infected and show no pathology but can be distinguished, in terms of cytokine expression pattern, from uninfected controls.

## Background

Paratuberculosis (Johne's disease) is a chronic intestinal disease of ruminants caused by the bacterium *Mycobacterium avium *subspecies *paratuberculosis*. The disease is responsible for extensive economic losses worldwide related to fatality and loss of productivity [1,2]. The route of disease transmission is mainly faecal-oral; and neonates, when not infected congenitally are infected by ingestion of bacteria from infected teats or the pasture transmitted via the dissemination of diarrhoea from infected animals [3]. Consequently the majority of animals within an infected herd or flock become infected [3]. However, in sheep infection can give rise to three different forms of disease with only about 30% of animals in an infected flock becoming clinically affected. The majority of animals are asymptomatic; they are infected but show no pathology and do not develop clinical disease [4]. The remaining clinically-affected sheep show two distinct forms of the disease. Approximately 30% of disease cases are affected by the paucibacillary form of Johne's disease; they have very few bacteria and show a T cell infiltration into the gut. About 70% of cases have the multibacillary form, which is characterized by a high level of bacterial infection and a macrophage and B cell infiltration into the gut lamina propria. Both the pauci- and multibacillary forms are equally fatal but there is no evidence that the asymptomatic animals ever succumb to disease [4,5].

Johne's disease pathogenesis in cattle may differ from that in sheep as it is presumed that there is disease progression from the asymptomatic to paucibacillary and then to the fatal multibacillary form [6]. However, even in cattle 4–11% of animals in affected herds are infected but only 2–4% develop the disease [7,8]. Like the two other major mycobacterial diseases, tuberculosis and leprosy, the epidemiology of paratuberculosis suggests a genetic susceptibility [9]. The paucibacillary cases (tuberculoid pathology) tend to have a strong cell-mediated immunity (CMI), high levels of IFNγ and IL-2 and low levels of antibody [10-12]; the multibacillary cases (lepromatous pathology) have high antibody and pro-inflammatory cytokine levels and weak CMI [10,12]. Little is known about the asymptomatic form although they are positive for bacterial growth, IS900 and specific antibody [13,14].

As with tuberculosis and leprosy it seems clear that the polarization of the immune response is critical to the clinical outcome of the paratuberculosis infection [15-17]. The intestinal tissue damage that results from a Th1 response (paucibacillary disease) is fundamentally different to that caused by a Th2 response, which leads to multibacillary disease and dissemination of infection. The polarization of the immune response is controlled by the differential expression of T cell polarizing cytokines [18] and it seems that there is differential expression of some of these cytokines in Johne's disease [19]. Several studies describe the changes in expression of cytokines in peripheral blood cells in cattle [20,21] and ileal tissue of both sheep [11,12] and cattle [19,21-24]. Burrells el al. (1999) [11] examined IL-2 and IFNγ in both pauci- and multibacillary forms but not the asymptomatic animals while Tanaka et al. [19] quantified cytokines in granulomatous lesions during the preclinical stages of disease; all other studies on the target organ concentrated on analysis of the multibacillary form, including all studies on the ileal tissue in cattle.

This study tested the hypothesis that expression levels of a panel of thirteen cytokine and cytokine associated genes would be different at the site of infection in the three forms of sheep paratuberculosis, and that these differences could relate to the observed pathologies. Furthermore, this study also analyses the asymptomatic form of sheep Johne's disease in relation to uninfected control animals.

## Results

### Definition of pathological forms

**Figure 1:**
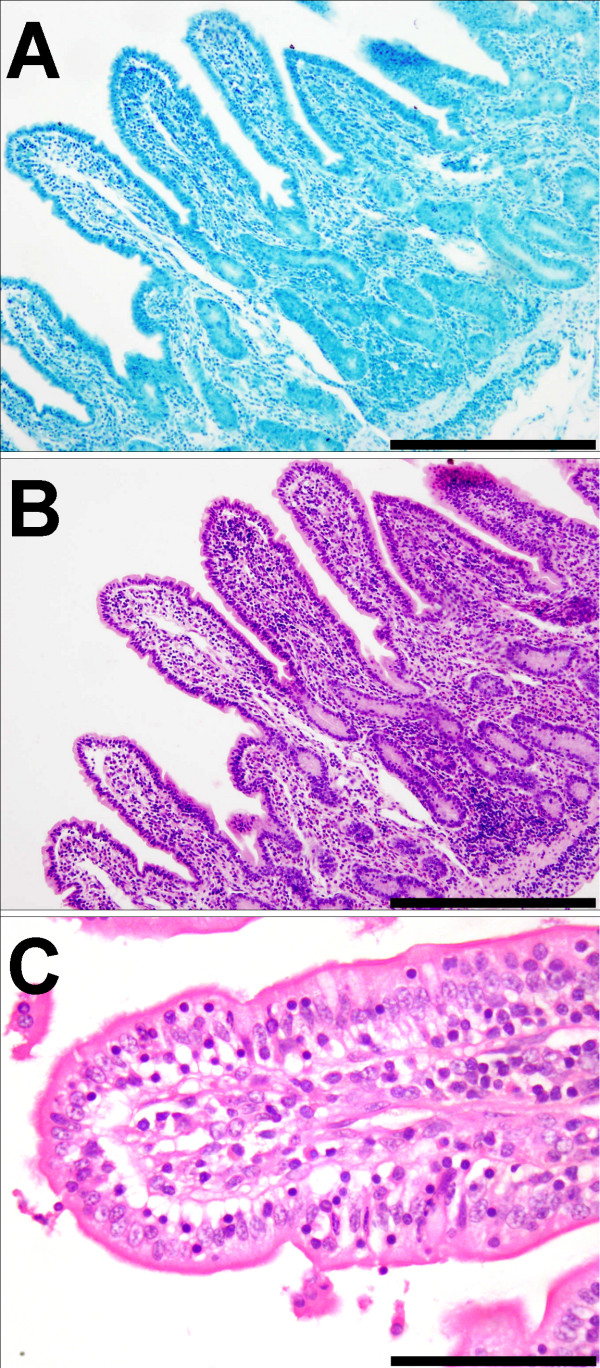
**Histopathology of the terminal ileum from asymptomatic sheep**. (a) Ziehl-Neelsen stain for acid-fast bacteria (× 250) showing the absence of mycobacteria. (b) H&E stained low power (×250) and (c) high power (×400) show normal histology.

**Figure 2:**
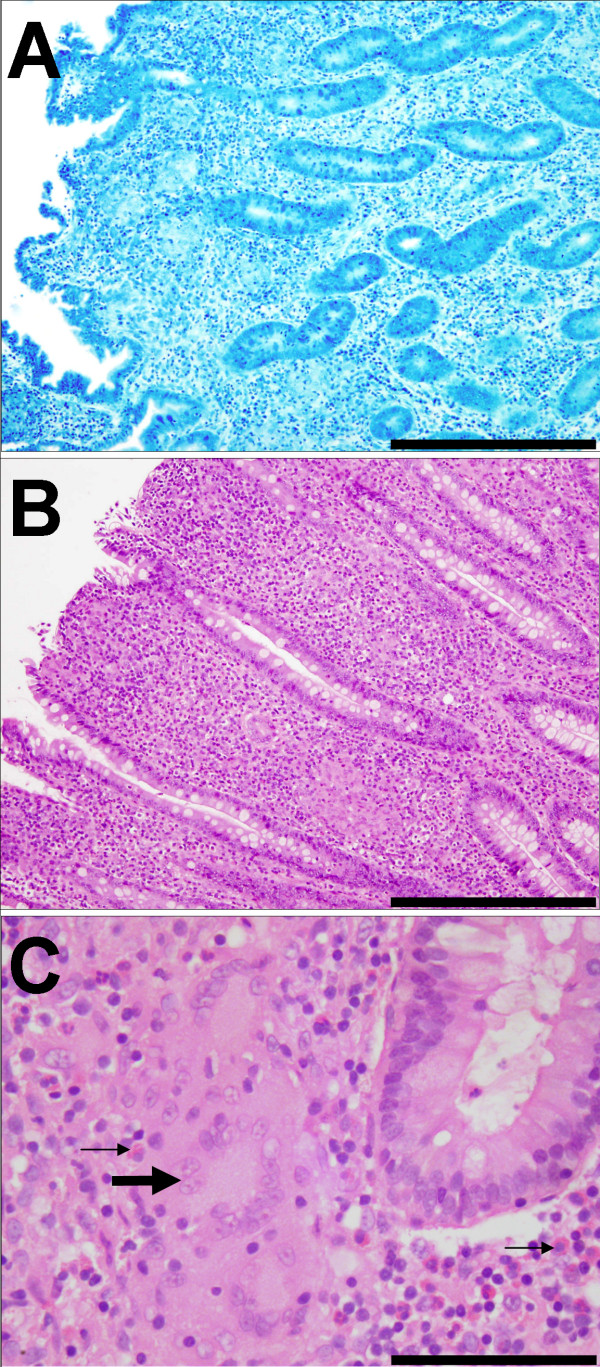
**Histopathology of the terminal ileum from paucibacillary sheep**. (a) Ziehl-Neelsen stain (× 250) showing the absence of mycobacteria. (b) H&E stained low power (×250) showing mixed inflammatory infiltrate into lamina propria comprising lymphocytes, eosinophils, macrophages and multinucleate giant cells. (c) H&E stained high power (×400) showing multinucleate giant cells (large arrow) adjacent to a crypt. There is an associated proprial inflammatory reaction dominated by lymphocytes and eosinophils (small arrows).

**Figure 3:**
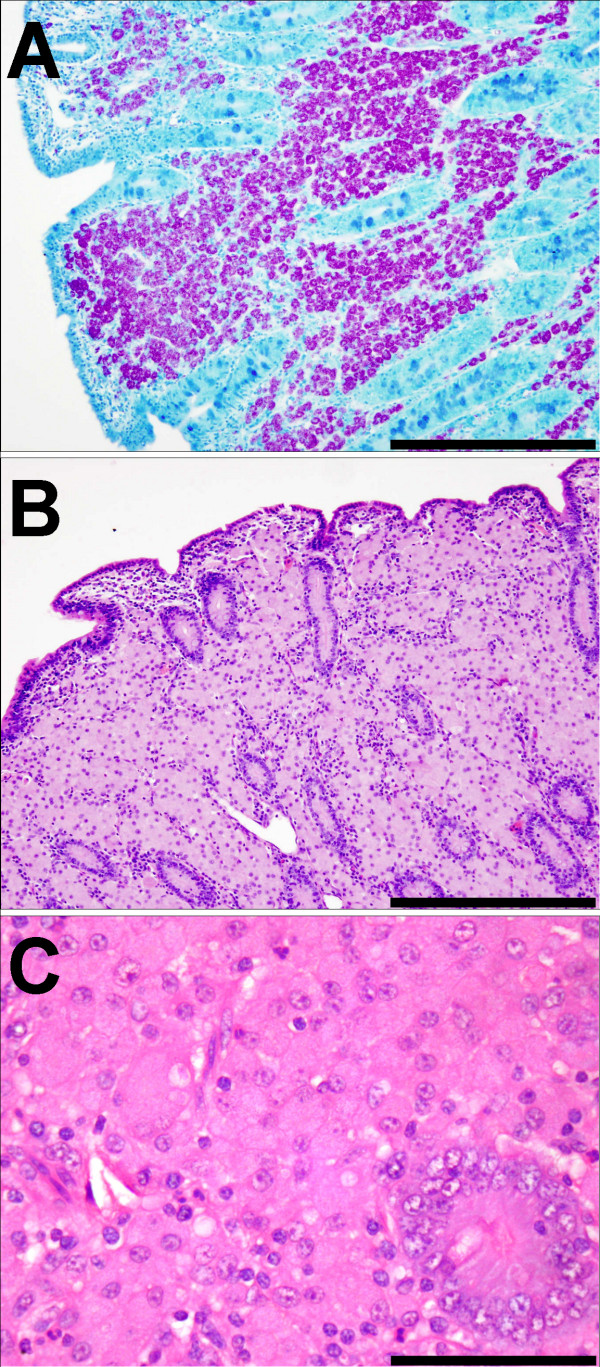
**Histopathology of the terminal ileum from multibacillary sheep**. (a) Ziehl-Neelsen stain (× 250) showing the presence of many intracellular mycobacteria associated with the infiltrating macrophages. (b) H&E stained low power (×250) showing infiltration of the lamina propria by sheets of epithelioid macrophages distending the propria and flattening the surface mucosa. (c) H&E stained high power (×400) demonstrating the uniform population of epithelioid macrophages with abundant cytoplasm infiltrating around an intestinal crypt.

All animals from the Johne's disease infected flocks were identified as IS900 positive by PCR analysis and therefore judged to be infected with MAP. Three groups of IS900+ animals could be discriminated on the basis of gross pathology, ZN staining and histopathology. The asymptomatic group had no clinical signs or lesions consistent with Johne's disease, at post mortem. Examination of histological sections of the terminal ileum showed normal histology and no evidence of the presence of acid-fast bacteria (ZN-) (Figure 1A–1C). All other sheep showed clinical signs and post mortem lesions consistent with Johne's disease and could be further differentiated into two groups (paucibacillary and multibacillary) on the basis of ZN staining of histological sections of terminal ileum. The paucibacillary sheep had no detectable ZN+ bacteria (Figure 2A) and showed a mixed inflammatory infiltrate into the lamina propria (Figure 2B) comprising lymphocytes, eosinophils and multinucleate giant cells (arrowed) with fewer macrophages (Figure 2C). The multibacillary sheep had high numbers of ZN+ acid-fast bacteria (Figure 3A), mostly within the cytoplasm of the large numbers of epithelioid macrophages, which distend the lamina propria (Figure 3B and 3C) and result in flattening of the surface mucosa (Figure 3B). A fourth group of unrelated, control sheep were IS900 negative and were judged to be uninfected with MAP.

### Gene expression analysis in ileal tissues

The expression levels of cytokine transcripts in sheep ileum from the three groups of paratuberculosis-infected sheep and in uninfected controls is shown in Table 1 which shows transcript copy number in relation to the two housekeeping genes, GAPDH and SDHA. These data show that different cytokines are present at very different levels in ileal tissue, varying from less than 100 copies (e.g. IL-3) to greater than 100,000 copies (e.g. IL-8 and IL-10). They also show that levels of individual cytokine transcripts show a high variability between animals, even within the same pathological group.

**Table 1:** Cytokine transcript levels in terminal ileum from sheep with paratuberculosis infected sheep and controls

**Gene**	**Multibacillary**	**Paucibacillary**	**Asymptomatic**	**Control**
IL-1α	69^1 ^± 56	38 ± 14	51 ± 42	20 ± 8
IL-1β	30839 ± 41353	16470 ± 8559	4176 ± 2557	8613 ± 6090
IL-3	29 ± 74	1 ± 3	87 ± 131	0 ± 0
IL-6	71778 ± 124310	11023 ± 2980	4996 ± 1698	5473 ± 7583
IL-8	1448098 ± 1453346	609805 ± 511880	480573 ± 601312	150388 ± 59472
IL-10	305964 ± 127868	174186 ± 82536	126941 ± 94659	240619 ± 185599
IL-12	7178 ± 7381	7589 ± 6467	4506 ± 5179	1615 ± 2436
IL-18	102838 ± 45161	84037 ± 34888	42352 ± 10066	126047 ± 41771
GM-CSF	2512 ± 1067	2973 ± 963	1786 ± 684	1986 ± 552
IFNγ	3354 ± 1063	5209 ± 2554	2529 ± 1620	1537 ± 427
TGF-β	50349 ± 30161	28007 ± 13809	21871 ± 11222	14519 ± 6032
TNFα	21113 ± 13334	11681 ± 7914	7492 ± 4925	2770 ± 2120
TRAF-1	11504 ± 6076	20087 ± 10563	5018 ± 5134	3708 ± 1662

^1^Copy number ± SD, normalized to SDHA and GAPDH

Furthermore, it shows that individual cytokines are differentially expressed in the distinct disease states.

**Figure 4:**
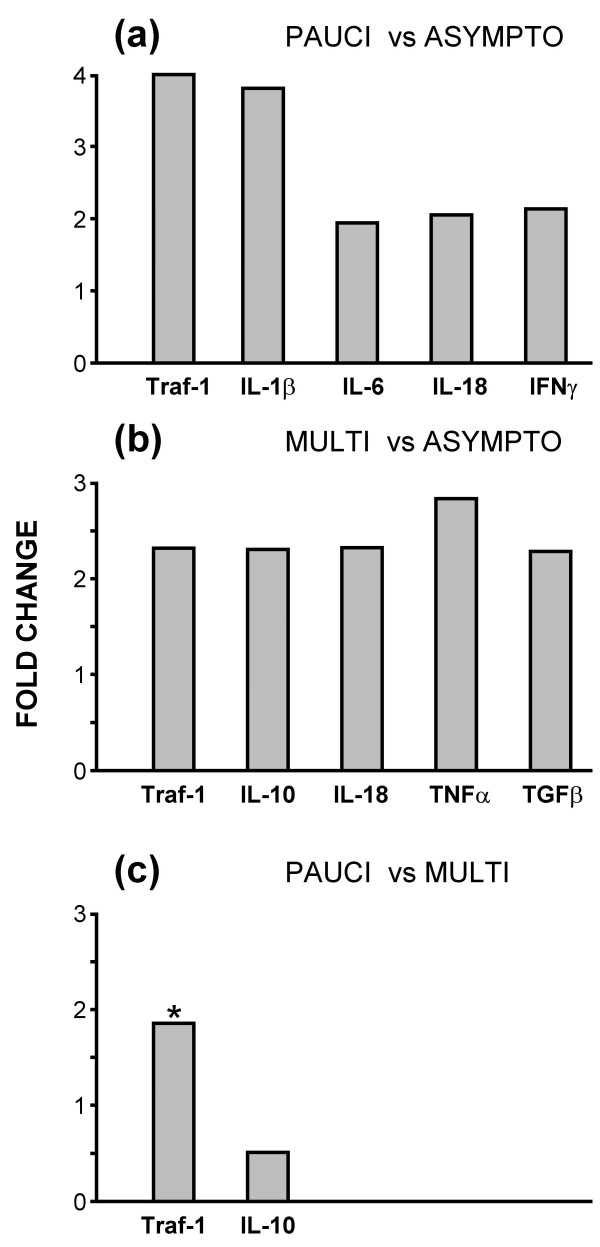
**Statistically significant changes in genes between the three IS900+ groups**. (a) Comparison of paucibacillary and asymptomatic; (b) comparison of multibacillary and asymptomatic; (c) comparison of paucibacillary and multibacillary. Results are given as significant (p ≤ 0.05) fold-changes of mean copy-numbers relative to the mean copy-numbers of the comparative group. IL-3 was not included, as the copy number in the control samples was 0, thus fold change values could not be calculated. The data for TRAF-1, highlighted with * in (c) excludes a single outlier in the ten data points; p = 0.062 with the outlier; p ≤ 0.02 without the outlier.

**Figure 5:**
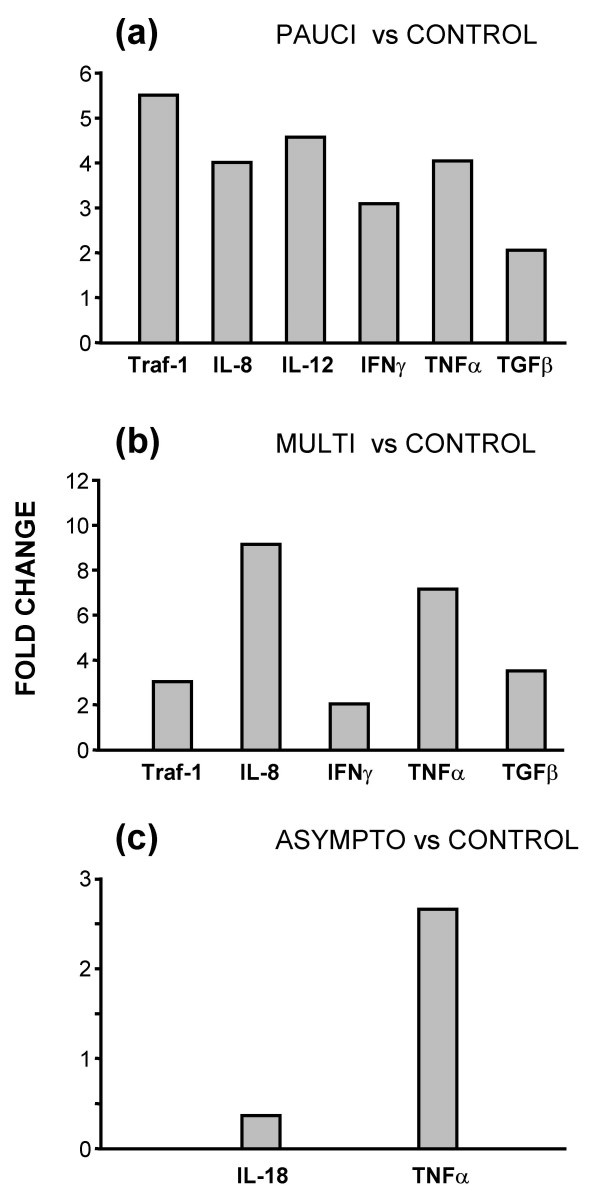
**Statistically significant changes in genes between the three IS900+ groups and the uninfected control group**. (a) Comparison of paucibacillary and control; (b) comparison of multibacillary and control; (c) comparison of asymptomatic and control. Results are given as significant (p ≤ 0.05) fold-changes of mean copy-numbers relative to the mean copy-numbers of the comparative group.

This is more clearly shown in Figures 4 and 5 where the results are expressed as statistically significant (p = 0.05) fold change where data are compared in six pairs – paucibacillary vs asymptomatic, multibacillary vs asymptomatic, paucibacillary vs multibacillary (Figure 4), paucibacillary vs control, multibacillary vs control and asymptomatic vs control (Figure 5). The expression levels of two genes, IL-1α and GM-CSF was relatively consistent in all the animals regardless of disease status.

Figure 4 shows the comparison of cytokine transcripts between the three infected groups of sheep. When the two diseased forms were compared with the asymptomatic samples TRAF-1, IL-1β, IL-6, IL-18 and IFNγ were significantly up-regulated in paucibacillary ileum, (Figure 4a) and TRAF-1, IL-10, IL-18, TNFα and TGFβ were up-regulated in multibacillary samples (Figure 4b). Comparison of the pauci- and multibacillary forms showed that only IL-10 was significantly different, with the expression levels in the paucibacillary ileum being approximately half that in multibacillary tissue. In contrast the level of IFNγ and TRAF-1 were much higher in paucibacillary tissue, however neither was quite significant (p = 0.057 and 0.062 respectively). If one outlier point in the TRAF-1 data was removed from the ten samples the significance level was increased to p = 0.02 (Figure 4c).

Figure 5 shows the comparison of cytokine transcripts between the three infected groups and the uninfected control animals. TRAF-1, IL-8, IL-12p40, IFNγ, TNFα and TGFβ were significantly increased in paucibacillary samples (Figure 5a). The multibacillary sheep showed significantly increased levels of TRAF-1, IL-8, IFNγ, TNFα and TGFβ (Figure 5b). The multibacillary ileum also showed increased levels of IL-12p40 but this was not quite statistically significant (p = 0.06). Interestingly there were significant differences between the asymptomatic and uninfected control samples, which showed that IL-18 was down-regulated and TNFα was up-regulated in the asymptomatic sheep. IL-3 was detected at low levels in all infected animals but never in uninfected control samples; therefore it was not possible to produce fold-change figures for these comparisons. Nevertheless, IL-3 was statistically significantly up-regulated (p = 0.05) in both multibacillary and asymptomatic samples (but not paucibacillary sheep) compared to controls, using the non-parametric Mann-Whitney test.

## Discussion

The three major mycobacterial diseases of mammals – tuberculosis, leprosy and paratuberculosis affect different organ systems but share at least two important characteristics. Firstly, not all infected individuals become clinically affected and second, there are at least two pathological forms of each disease [2,25,26]. This is most obvious in human leprosy where, although there is a spectrum of disease, the majority of patients are at either end of that spectrum [27]. The tuberculoid form of the disease has lesions formed of infiltrating T cells and contain few bacteria; and the lepromatous form has lesions of infiltrating macrophages containing large numbers of bacteria [27,28]. The pathological picture of ruminant paratuberculosis seems to mimic human leprosy [29] although this is most true of the disease in sheep where the pathology can be differentiated into tuberculoid (paucibacillary), lepromatous (multibacillary) and asymptomatic (infected but no pathology) [1]. There is a difference between these two diseases however as the sheep tuberculoid form is an end stage disease [5] and it is not 'self-curing' as it is in leprosy [27]. There also seem to be differences between ovine and bovine paratuberculosis, as in cattle the pathological form seems to be related to the disease development [6,29], with early stage tuberculoid lesions developing into end-stage lepromatous disease. In addition, intermediate stages between these two extremes have also been reported [30,31]. Similar disease patterns are also observed in tuberculosis, but as with bovine paratuberculosis they seem to be more variable than leprosy [32].

Immunological responses to infectious disease challenge have a major bearing on the clinical manifestations of that infection and the clinical consequence for the host. This is most obvious in human leprosy [33,34] where the type 1 T cell cytokine pattern predominates in the self-curing tuberculoid patients in contrast to the type 2 pattern in malign lepromatous disease. The aim of this present study is to try and understand the relationship between the immunology and the different pathological changes that occur as a result of *Mycobacterium avium *subspecies *paratuberculosis *infection in sheep.

Despite the fact that there is a large variation in the levels of cytokine transcript expression, this study shows unambiguously that there is a relationship between the three pathological forms of sheep paratuberculosis and the immune response as represented by the cytokine transcript profiles within the target tissue. The large variation is probably due to the fact that these sheep are unrelated, of different breeds and are infected naturally rather than experimentally; in addition they carry an unquantified gastrointestinal worm burden that might influence ileal cytokine expression. We cannot measure levels of functional protein for all the cytokines within the ileum but assume that the relative quantities of cytokine protein and transcript are linked.

As has been shown with bovine paratuberculosis [19-21], the paucibacillary and multibacillary forms of the sheep disease are also associated with the polarization of the immune response. The comparison of each form of the disease with tissue from asymptomatic animals shows that IFNγ is significantly increased in the paucibacillary lesions and IL-10 is raised in the multibacillary form. Furthermore, direct comparison of the diseased tissues shows that these two cytokines are reciprocally expressed, although the increase (x1.55 fold) of IFNγ in paucibacillary ileum is not quite significant (p = 0.057). This confirms that in sheep paratuberculosis polarized type 1 and type 2 T cells are strongly associated to paucibacillary and multibacillary pathology respectively. The major biological function of IL-18 seems to be linked to inducing type 1 responses and stimulating IFNγ production [35]. However, the pattern of IL-18 expression is not explained simply in terms of type 1 and type 2 responses as it is up-regulated in both disease forms. Perhaps the increased TGFβ level in the multibacillary ileum explains the low IFNγ levels in that tissue [36].

Comparison of both disease forms to uninfected control samples shows up-regulation of TRAF-1, IL-8, IFNγ, and TNFα, reflecting the high levels of inflammation present in the affected tissues and agrees with the data in cattle [21,24]. TRAF-1 is anti-apoptotic and its up-regulation in both disease forms indicates that it may be associated with the accumulation of several different cell types in the lamina propria and not just macrophages [24]. Previous studies in stimulated PBMC from infected cattle have shown down-regulation of IL-12p35 in infected cattle compared to controls [21]. However, this study showed significant (p = 0.05) up-regulation of IL-12p40 in paucibacillary samples (p = 0.06 for multibacillary tissue). This may be explained by differences in the tissues tested or that IL-12p40 levels reflect IL-23 and not IL-12p70. IL-1β and IL-6 were also significantly raised in paucibacillary ileum compared to asymptomatic samples, the increase in multibacillary samples was large but without statistical significance (p = 0.12). Both have been shown previously to be up-regulated in multibacillary disease [37], but as with IL-8 and TNFα they are also associated with general inflammation. Unlike other gastrointestinal inflammatory lesions [38], the expression of large quantities of pro-inflammatory cytokines in paratuberculosis is not reflected in the large scale infiltration of neutrophils. A possible explanation for this is the presence of increased levels of TGFβ, which has known anti-inflammatory and immunosuppressive functions [36]. IL-3 is absent from uninfected ileum but can be detected in both asymptomatic and multibacillary tissues; however, the low levels of transcripts present leave doubt about its biological relevance. Our data only partly fit with the 'Coussens' model of bovine paratuberculosis pathogenesis [39]. They are almost identical in relation to the T cell polarizing cytokines and most of the inflammatory mediators but contrasts in relation to IL-1α. This cytokine is highly up-regulated in infected bovine tissue and the model hypothesises that the pathogenesis of paratuberculosis may be partly due to IL-1α toxicity. However our data show that IL-1α is virtually absent from sheep ileum. Apart from species differences a possible explanation for this discrepancy is that all the sheep paratuberculosis tissues originate from terminal disease states and not during the development of the pathology.

Of particular interest is the comparison between the infected, asymptomatic and the uninfected, control sheep. Unlike cattle, where asymptomatic animals are regarded as 'preclinical' and show histological lesions [19], asymptomatic sheep show completely normal ileal histology and their susceptibility to disease has been questioned [4]. The normality of the asymptomatic cases is largely confirmed by the fact that the majority of cytokines are expressed at 'normal' levels; however TNFα and IL-18 are exceptions, TNFα is significantly up-regulated and IL-18 is down-regulated in asymptomatic samples. The low levels of IL-18 are not matched by a similar down-regulation of IFNγ.

## Conclusion

In this study, we have quantified the expression levels of thirteen important immunoregulatory genes in the three forms of ovine paratuberculosis using real-time RT-PCR analyses. We have confirmed that the paucibacillary and multibacillary pathologies are linked to type 1 and type 2 T cell responses respectively. However, many other cytokines are differentially expressed and it is clear that these cytokines are part of a complex network and interact to form the final pathologies. It is also clear that asymptomatic animals are also responding to infection, although the data do not explain how the disease is controlled in these animals. The epidemiology of the disease would suggest a genetic susceptibility similar to that recognised for tuberculosis and leprosy, and these data suggest several likely candidates for future genetic analysis.

## Methods

All procedures involving animals was approved by the Home Office under the appropriate Animals (Scientific Procedures) Act 1986 Project Licence.

### Experimental animals/tissues

Animals with the clinical disease and asymptomatic animals were out bred sheep with naturally acquired MAP infection from one of three farms (Table 2). Individual sheep were delivered to the Moredun Research Institute based on clinical symptoms of Johne's disease. All sheep were euthanized and the diagnosis of Johne's disease was confirmed by histopathology of the terminal ileum and IS900 real-time PCR. Sheep from the same flocks with no clinical signs of Johne's disease but that were positive for IS900 were considered to be asymptomatic [4]. All control sheep tested negative for IS900. There were ten animals in each of the infected group and nine uninfected controls. Terminal ileum samples for histopathology were removed post-mortem and fixed in 10% formol-saline. Four μm sections from paraffin wax-embedded tissue were stained with haematoxylin and eosin or Ziehl-Neelsen (ZN). For RNA preparations, tissue blocks (~0.5 g) were placed in five volumes of in RNAlater (Ambion, Huntingdon, UK), which were then incubated overnight at 4°C and then stored at -80°C.

**Table 2:** Breed, age and sex of sheep

**Disease Type**	**Sheep Breed**	**Age (years)**	**Sex**
Multibacillary	Blackface × Bleu du Maine	4.5	F
Multibacillary	Blackface × Bleu du Maine	4.5	F
Multibacillary	Blackface	5	F
Multibacillary	Blackface	5	F
Multibacillary	Blackface	3	F
Multibacillary	Blackface	3	F
Multibacillary	Blackface	2	F
Multibacillary	Blackface	4	F
Multibacillary	Blackface	4	F
Multibacillary	Blackface	3	F
			
Paucibacillary	Blackface × Bleu du Maine	4.5	F
Paucibacillary	Texel	5.5	F
Paucibacillary	Blackface × Bleu du Maine × Lleyn × Roussin	2.5	F
Paucibacillary	Lleyn × Roussin	3.5	F
Paucibacillary	Blackface × Bleu du Maine	4.5	F
Paucibacillary	Blackface × Bleu du Maine	4.5	F
Paucibacillary	Bleu du Maine	4	F
Paucibacillary	Blackface × Bleu du Maine	6	F
Paucibacillary	Texel	2.5	F
Paucibacillary	Blackface	2.5	F
			
Asymptomatic	Blackface × Bleu du Maine	4.5	F
Asymptomatic	Blackface × Bleu du Maine	4.5	F
Asymptomatic	Blackface × Bleu du Maine	7	F
Asymptomatic	Blackface × Bleu du Maine	7	F
Asymptomatic	Texel	1	F
Asymptomatic	Blackface	2.5	F
Asymptomatic	Greyface	3	F
Asymptomatic	Greyface	3	F
Asymptomatic	Greyface	3	F
Asymptomatic	Greyface	3	F

The uninfected controls were: 6 Blackface (F) aged 4 years, 3 Texel (F) aged 4/5 years.

### IS900 quantitative PCR

All RNA samples were tested for the presence of MAP by IS900 real-time RT-PCR for a MAP specific insertion sequence [40]. Primers and probes were as Eishi et al. [41], and the reactions were carried out in an ABI Prism 7000 real-time PCR machine. Each reaction contained 12.5 μl Platinum^® ^Quantitative PCR SuperMix-UDG with ROX (Invitrogen; Paisley, UK), 50 nM each primer, 100 nM probe and 5 μl cDNA, made up to 25 μl with deionised water. The reactions were cycled as follows: 50°C for 2 minutes, 95°C for 10 minutes and 40 cycles of 95°C for 15 seconds, then 60°C for 1 minute.

### Isolation RNA Isolation and cDNA synthesis

Tissue samples were thawed at room temperature, 250 mg wet weight of ileum tissue was shredded and then disrupted and homogenised in a buffer containing 4 M guanidine isothiocyanate using a ribolyser (Fast-Prep from Thermo-Hybaid, Runcorn, UK). Total RNA was extracted using a Qiagen RNeasy^® ^Maxi Kit (Qiagen, Crawley, UK) and DNase treated using the Ambion DNase I kit (Ambion, Huntingdon, UK). Samples were eluted into 800 μl of nuclease-free water. The RNA was quantified by spectrophotometry, ethanol precipitation and resuspended in 300 μl of nuclease-free water. Samples were checked for genomic DNA contamination by GAPDH PCR.

For cDNA synthesis, 2.5 μg of RNA was diluted to a final volume of 16.25 μl in nuclease free water. 500 ng of Oligo (dT) (Promega, Southampton, UK) was added, and incubated for five minutes at 70°C, followed by five minutes on ice. To this 5 μl of 5× MMLV buffer, 1.25 μl of 100 mM dNTPs and 1 μl of MMLV RT enzyme (Promega) were added, and the mixture was incubated at 42°C for 60 minutes, followed by 15 minutes at 70°C to inactivate the enzyme. The cDNA was diluted four-fold in nuclease free water and stored at -20°C until used.

### Cloning of ovine cytokine gene fragments

Primers (Table 3) were selected and designed using Primer3 [42] software. All selected primer sequences were then checked for possible cross-hybridization using the BLAST [43] and subjected to quality check using Net Primer [44]. The PCR mixture contained 2 μl of cDNA; 5 μl (10×) PCR buffer (Promega);1 μl dNTP mix (Promega); 20 pmol of each primer (Sigma-Genosys, Haverhill, UK);1 μl *Taq* polymerase (5 units) and nuclease free water was added to a final volume of 50 μl. Reactions were then cycled under the following conditions: 95°C for 2 mins 30 cycles of 30 s at 95°C, annealing at (see Table 1), 45 s; 60 s at 72°C, followed by a final extension at 72°C for 5 mins. PCR products were analysed by agarose gel electrophoresis, visualized by ethidium bromide/UV transillumination, purified using the QIAquick^® ^system (Qiagen), cloned into pGEM T-Easy^® ^(Promega) and sequenced.

**Table 3:** Primer sequences used for real time RT- PCR

**Gene Accession number**	**Primer Sequence 5'-3'**	**Product size (bp)**
IL-1α GenBank: NM_174092	**F: **TTGGTGCACATGGCAAGTG **R: **GCACAGTCAAGGCTATTTTTCC	72
IL-1β GenBank: X56972	**F: **CCTTGGGTATCAGGGACAA **R: **TGCGTATGGCTTTCTTTAGG	317
IL-3 GenBank: Z18897	**F: **ACCTCCTTCTGCTCCTGCTT **R: **TATTCCCAAGTCCCCATCTT	193
IL-6 GenBank: X68723	**F: **TCCAGAACGAGTTTGAGG **R: **CATCCGAATAGCTCTCAG	236
IL-8 GenBank: X78306	**F: **ATGAGTACAGAACTTCGA **R: **TCATGGATCTTGCTTCTC	222
IL-10 GenBank: U11421	**F: **CTGTTGACCCAGTCTCTGCT **R: **ACCGCCTTTGCTCTTGTTT	305
IL-12p40 GenBank: AF004024	**F: **TCAGACCAGAGCAGTGAGGT **R: **GCAGGTGAAGTGTCCAGAAT	243
IL-18 GenBank: AJ401033	**F: **GAGCACAGGCATAAAGATGG **R: **TGAACAGTCAGAATCAGGCATA	241
IFNγq GenBank: X52640	**F: **CTAAGGGTGGGCCTCTTTTC **R: **CATCCACCGGAATTTGAATC	55
TNFα GenBank: X56756	**F: **GAATACCTGGACTATGCCGA **R: **CCTCACTTCCCTACATCCCT	238
TGFβ GenBank: X76916	**F: **GAACTGCTGTGTTCGTCAGC **R: **GGTTGTGCTGGTTGTACAGG	169
GM-CSF GenBank: X53561	**F: **GATGGATGAAACAGTAGAAGTCG **R: **CAGCAGTCAAAGGGAATGAT	261
TRAF1 GenBank: XM_589090	**F: **AGCAGAGGGTGTTGGAGTTG **R: **CTGGGGAGAAGAGGCTGAC	186
GAPDH GenBank: AF030943	**F: **GGTGATGCTGGTGCTGAGTA **R: **TCATAAGTCCCTCCACGATG	265
SDHA GenBank: NM_174178	**F: **ACCTGATGCTTTGTGCTCTGC **R: **CCTGGATGGGCTTGGAGTAA	126

### Quantitative real-time PCR

Two-step, quantitative real-time RT-PCR was carried out using a Rotor-Gene™ 3000 (Corbett Life Science, Cambridge, UK) using primers as in Table 3. Standard curves for each gene were generated using 10-fold serial dilution series of linearized plasmid DNA templates. Quantitative real-time PCR reactions were run in 20 μl containing 2 μl of FastStart Taq buffer, 200 μM dNTPs (Promega), 250 nM each primer, MgCl2 to an optimum concentration, 0.7 μl of a 1/1000 dilution of SYBR green master mix, 0.75 U FastStart Taq DNA Polymerase(all Roche Diagnostics, Lewes, UK) and 2 μl of template cDNA, made up to 20 μl with deionised water. The cycling conditions for all genes were as follows: 5 minutes at 94°C, 45 cycles of 20 seconds at 94°C, 20 seconds at 60°C and 20 seconds at 72°C, followed by a melt curve starting at 65°C rising to 94°C at 0.3°C per second. Copy numbers were determined from the Ct values of each sample in comparison to the copy number values assigned from the plasmid DNA standard using Rotor-Gene analysis software (6.0.34). Data were normalized using glyceraldehyde-3-phosphate dehydrogenase (GAPDH) or succinate dehydrogenase (SDHA) housekeeping genes. A normalization factor was calculated taking into account the 75th percentile of the housekeeping gene copy numbers for each run. Results were compared pair wise using a 2-sample t test to determine statistical significance. Each sample was analysed in duplicate, n = 10 for each IS900+ sheep and n = 9 for the uninfected controls.

### Variability assay

To determine the level of variability inherent in the real-time PCR reactions, a variability assay was carried out. A single sample was reverse transcribed in three simultaneous reactions. The cDNA produced was then amplified ten times each in an SDHA real-time PCR reaction. The resulting copy numbers were compared to give a value for the variability inherent within the reactions. The assay showed that the overall variability inherent to the method is 2.2 fold.

## Competing interests

The authors declare that they have no competing interests.

## Authors' contributions

JAS performed the real-time PCR experiments and was responsible for the draft manuscript preparation. CAW supervised JAS in the practical work; he performed the post-mortems and helped JAS with data analysis and draft manuscript preparation. SMR performed the histopathological diagnosis and analysis. JH was in overall control of the project and was responsible for its design, coordination and funding; he produced the final manuscript.
